# Postural control among individuals with and without chronic obstructive pulmonary disease: A cross-sectional study of motor and sensory systems

**DOI:** 10.1371/journal.pone.0284800

**Published:** 2023-04-25

**Authors:** Viktor Strandkvist, Anne Lindberg, Agneta Larsson, Mascha Pauelsen, Caroline Stridsman, Lars Nyberg, Helena Backman, Ulrik Röijezon

**Affiliations:** 1 Division of Health, Medicine and Rehabilitation, Department of Health, Education and Technology, Luleå University of Technology, Luleå, Sweden; 2 Department of Public Health and Clinical Medicine, Section of Medicine, Umeå University, Umeå, Sweden; 3 Department of Public Health and Clinical Medicine, The OLIN Unit, Section of Sustainable Health, Umeå University, Umeå, Sweden; Liverpool John Moores University, UNITED KINGDOM

## Abstract

**Background:**

Chronic obstructive pulmonary disease (COPD) is considered a heterogenic syndrome with systemic effects, including muscle dysfunction. There is evidence of postural control impairments among individuals with COPD, partly related to muscle weakness. However, research is scarce regarding the other underlying systems of postural control, such as the visual, somatosensory and vestibular system. The aim was to compare postural control, as well as the motor and sensory systems, between individuals with and without COPD.

**Methods:**

Twenty-two participants with COPD (mean age 74.0 ±6.2 years) and 34 non-obstructive references (mean age 74.9 ±4.9 years) participated in this cross-sectional study. Postural control was assessed with center of pressure trajectory of postural sway in quiet as well as a limits of stability test, calculating mediolateral and anteroposterior amplitudes for each test. Assessment of function in the motor system included maximum hand grip strength, as well as maximum strength in muscles around the hip, knee and ankle joints. Visual acuity, pressure sensibility, proprioception, vestibular screening, and reaction time were also included. Data was compared between groups, and significant differences in postural control were further analyzed with an orthogonal projection of latent structures regression model.

**Results:**

There was a significantly increased sway amplitude in the mediolateral direction in quiet stance on soft surface with eyes open (p = 0.014) as well as a smaller anteroposterior amplitude in the limits of stability test (p = 0.019) in the COPD group. Regression models revealed that the mediolateral amplitude was related to visual acuity and the burden of tobacco smoking assessed as pack-years. Further, muscle strength associated with anteroposterior amplitude in limits of stability test in the COPD group, and with age and ankle dorsal flexion strength among the referents. Besides for lower ankle plantar flexion strength in the COPD group, there were however no significant differences in muscle strength.

**Conclusions:**

Individuals with COPD had a decreased postural control and several factors were associated with the impairments. The findings imply that the burden of tobacco smoking and reduced visual acuity relate to increased postural sway in quiet stance, and that muscle weakness is related to decreased limits of stability, among individuals with COPD.

## Introduction

Chronic obstructive pulmonary disease (COPD) is an endemic disease estimated to affect between 8–10 percent of all adults [1, 2], and is considered a heterogenic syndrome with systemic effects [3]. Skeletal muscle dysfunction is common in COPD, including muscle weakness, muscle atrophy, mitochondrial dysfunction, poor oxidative capacity and a shift in muscle fiber type [4]. This dysfunction is contributing to exercise limitation and functional limitations including postural control [5], which may be related to the increased risk of falls in this population [6].

Postural control is a complex motor skill and acts in order to maintain balance and stability. It has influences from multiple motor and sensory operations and this ability involves activation of the central nervous system, combining information from visual, vestibular and somatosensory receptors along with motor actions and reactions [7]. Evidence are emerging of balance reductions in studies of individuals with moderate to very severe COPD compared to healthy references [8–11], and it seems that postural control in the mediolateral direction might be affected more than the anteroposterior [9, 12]. The incidence of falls is higher among individuals with severe COPD than individuals without COPD [6], and falls among older adults are associated with a decrease in functional independence, social interaction and life expectancy [13].

The cause of the postural control impairments in COPD is not fully understood, however, it is partly associated with muscle weakness [5]. Research on the other underlying systems of postural control besides muscle function is scarce. A study of individuals with moderate to severe COPD found impairments in postural control as well as lower muscle strength in the knee extensors compared to those without COPD [8]. The study included trials of quiet stance in different sensory conditions and challenged the postural control by altering the sensory input and integration from the visual, somatosensory, and vestibular systems. However, there were no group differences when analyzing the contribution of each system [8].

Although postural control has been studied during experimental perturbation of the various sensory systems [10, 11, 14], no previous study has, to the best of our knowledge, included specific tests of the various sensory systems to analyze their associations with postural control in COPD. This knowledge is important to further improve feasible and effective interventions [15] to prevent falls in this population.

The aims of this motion laboratory study were to compare 1) postural control, and 2) the motor and sensory systems involved in postural control, between individuals with and without COPD. A further aim was to 3) investigate the associations between reduced postural control and function of the motor and sensory systems in individuals with and without COPD.

## Methods

The study had a cross-sectional observational design where postural control and the motor and sensory systems were assessed, comparing individuals with and without COPD. The regional ethical review board in Umeå, Sweden, approved the study (2015/182-31 & 2017-313-32M).

### Participants

**Fig 1:**
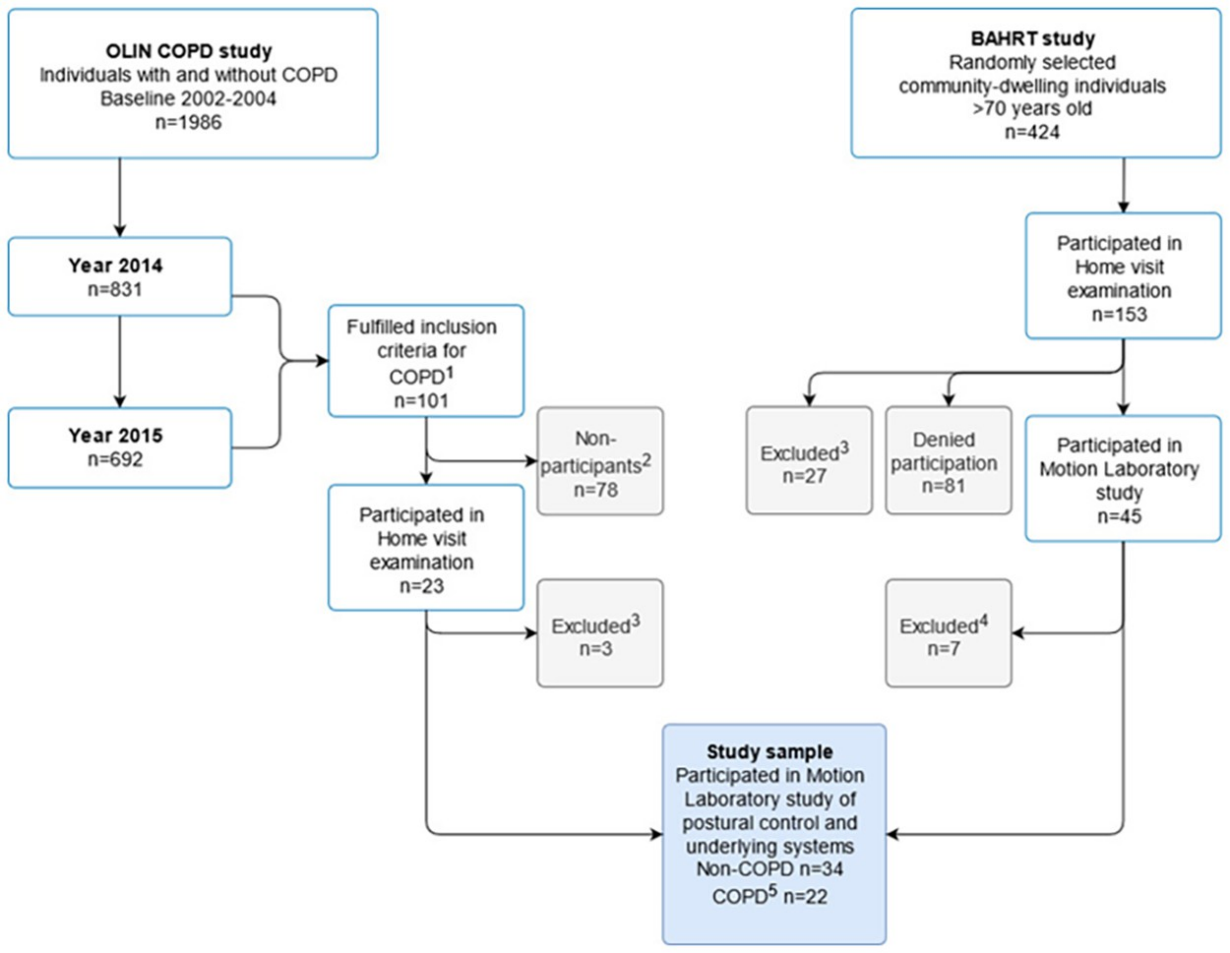
Flow chart over the recruitment of the study sample. **Notes**: ^1^Post bronchodilator FEV_1_/VC <0.70 & FEV_1_% of predicted<80%, >68 years old, living in Luleå, Boden or Piteå municipality. ^2^Individuals who denied participation or could not be located. ^3^ Individuals not fulfilling the additional inclusion criteria, including adequate vision to read 100 pt font size, ability to stand unassisted for at least 30 seconds, and to understand and process simple instructions. ^4^FEV_1_/VC <0.70, but did not perform post-bronchodilator spirometry. ^5^n = 20 from OLIN COPD study, n = 2 from BAHRT study.

Individuals with COPD were recruited from the longitudinal population-based Obstructive Lung disease In Northern Sweden (OLIN) COPD-study during 2014 or 2015 [16, 17] (Fig 1). Individuals were eligible for inclusion if they; 1) fulfilled the spirometric criteron for COPD according to the Global Initiative for Obstructive Lung Disease (GOLD) [18] stage ≥ 2 (post-bronchodilator FEV_1_/(F)VC<0.70 & FEV_1_<80% of predicted), 2) were aged 68 or older, and 3) lived in the municipalities of Luleå, Piteå or Boden. These individuals (n = 101) were contacted by telephone and informed of the study and invited to participate. In addition to the COPD group, a reference group without airway obstruction (FEV_1_/VC≥0.70) was included from the Balancing Human and Robot project (BAHRT), a previously conducted population-based study including 45 older adults (>70 years) in the same laboratory as the present study [19]. Two participants of this latter study fulfilled the spirometric criteria for COPD GOLD stage ≥ 2 and were thus included in the COPD-group (thereby, n = 22 in total). Seven participants had FEV_1_/VC<0.70, but did not perform a post-bronchodilator reversibility, and where thus excluded from the reference group (thereby, n = 34 in total) (Fig 1). All participants gave written informed consent before collection of any data.

### Data collection

Firstly, basic characteristics were collected during a home visit, including self-reported data on demographics, health, disease history, fall history, smoking habits and use of prescription medication. The home visit also included measurements of balance, gait and chair stands with the Short Physical Performance Battery (SPPB) (0–12) [20]. Secondly, the participants were invited to a laboratory visit where data from posturography and motor and sensory systems assessments as well as self-reported physical activity [21] were collected at the Human Health and Performance Lab–Movement Science at Luleå University of Technology, during 2016–2018. Additional inclusion criteria were used for the laboratory visits, including adequate vision to read 100 pt font size, ability to stand unassisted for at least 30 seconds, and to understand and process simple instructions. No other exclusion criteria were implemented, the sample was supposed to reflect all community-dwelling individuals in the municipality.

The participants were divided into non-smokers, ex-smokers (stopped since at least 12 months), and smokers. “Any respiratory symptom” was defined as at least one of persistent cough, productive cough, recurrent wheeze, or dyspnea. “Cardiovascular disease” was defined as at least one of coronary heart disease, congestive heart failure or stroke. “Health: better than other” was defined as self-related health score of better than others in same age.

#### Postural control

The postural control measurements have previously been described in detail [19]. Using a Kistler force plate (Kistler, Switzerland) Center of Pressure (CoP) trajectory of postural sway was measured during quiet stance during four different trials of 30 seconds each: stable firm surface with eyes open (SEO), stable firm surface with eyes closed (SEC), unstable soft surface with eyes open (UEO), and unstable soft surface with eyes closed (UEC). For the unstable soft surface, a balance pad of 6 cm thickness (AIREX, Switzerland) was placed on the force plate. The CoP was also measured during limits of stability (LoS)-test by asking the participant to lean as far as possible in the anteroposterior (AP) and mediolateral (ML) directions with eyes open on a stable firm surface without moving their feet. Stance width was standardized by placing the first metatarsal heads at a distance equal to 75% of the width between the anterior superior iliac spines. The force plate used in measurement of postural control generated 16 signals at 3000 Hz from which the CoP trajectories were derived. Thereafter a low-pass 4th order Butterworth filter with a cut-off at 10 Hz was used with MATLAB R2017a (MathWorks, USA) to apply and extract maximum AP and ML amplitude (max AP & max ML), which describes the participant’s CoP-excursions during the four quiet stance trials. A smaller amplitude equals smaller sway, and thereby better postural control. The maximum AP and ML amplitude measurements for the LoS test was also extracted (AP LoS & ML LoS) in the same manner. Here, a larger amplitude indicates better control of the center of mass over the base of support, thereby better postural control. All CoP data was normalized to height (CoP variable/body height) before analysis.

#### Motor system—Muscle strength

Maximum isometric strength of muscles in lower left limb were measured with a Biodex system 3 (Biodex, USA), which measured the maximum torque (Nm) generated by muscles around the hip (extension & abduction), knee (extension & flexion) and ankle joint (plantar & dorsal flexion) in a fixed position, in that order [22]. Three attempts were performed for each muscle group with the left side only, and the highest value was used for analysis. Each attempt lasted 5 seconds with 30 seconds of resting time. Maximum isometric hand grip strength (N) was measured with a hand-grip dynamometer (E-LINK, Biometrics, UK). The participants were instructed to sit down with their elbows flexed at an angle of 90 degrees, with the dynamometer held by the hand in a neutral position [23]. Three attempts were performed with each hand, and the highest value of both left and right hand, respectively, was used for analysis. Grip strength was measured before the postural control trials and the lower limb strength was measured after, to not induce fatigue. Force and torque data was normalized to body weight (force/body weight^0.67^, torque/body weight^1^) [24].

#### Sensory systems

The tests for the sensory systems are described in depth elsewhere [19]. Bi-ocular vision acuity was screened with an NFD vision chart with scores ranging from 0.1 to 2.0 with a higher score indicating better visual acuity. Frenzel glasses was used to test vestibular function, the occurrence of positive nystagmus was investigated in left to right head rotation at different levels of passivity and speed as well as glancing left, right, up, and down with head in a neutral position. The assessment was judged dichotomous as either negative or positive.

Assessment of somatosensory systems included tests of proprioception and tactile input of the lower extremity. Proprioception was assessed with active joint position sense test of the left knee and the left foot with the use of Biodex System 3. The target position for knee joint repositioning was 30° flexion, with starting position at 90°. The target position for ankle joint repositioning was 5° dorsal flexion, with starting position at 20° plantar flexion. The absolute error (AE) mean of three trials was used for analyzes and lower AE indicated better proprioception. Pressure sensibility around the ankles was assessed with monofilaments of different stiffness, pressed on the skin on the lateral malleoli (increments: 0.4, 2, 4, 10, 300 g). Starting with the lightest, each monofilament was tested three times on each malleolus until the participant felt the touch of the filament, lower values indicated better pressure sensibility.

#### Reaction time

A custom made reaction time test (RT) on the laboratory computer was used to test reaction time. At random time intervals between 5 and 10 seconds, the participant had to push a button as fast as possible when a visual and audio cue was produced. The average of five attempts was used and smaller score indicated better reaction time.

#### Spirometry

The spirometry was performed using an ultrasonic flow spirometer, EasyOne (NDD, Switzerland), according to standards set by European Respiratory Society/American Thoracic Society [25]. COPD was defined as FEV_1_/(best of SVC or FVC) <0.70, and FEV_1_% of predicted <80%, using the highest value pre- or post bronchodilation. Reversibility testing was performed if FEV_1_/(best of SVC or FVC) <0.70 or FEV_1_ <80% of predicted, after inhalation of 2x0.2mg of salbutamol. Non-COPD was defined as FEV_1_/ (best of SVC or FVC) ≥ 0.70. The severity of obstruction was based on FEV_1_% of predicted. The OLIN reference values for spirometry were used [26]. The spirometry, including inhalation of salbutamol, was performed last to not interfere with the assessments of motor and sensory systems.

### Statistical analysis

All statistical analyzes were conducted with SPSS for Windows 24.0 (SPSS Inc., USA) or SIMCA 14.0 (Umetrics AB, Sweden). SPSS was used to analyze differences in proportions with Chi-2 test, differences in mean values between groups with two-tailed student’s t-test, and differences in mean ranks in non-normally distributed data with two-tailed Mann-Whitney u-test. All comparisons between the COPD and reference group regarding muscle strength and postural control were adjusted for age and sex, using analyzes of variance (ANCOVA). P-values <0.05 were considered statistically significant. Postural control variables that were significantly different between the groups were further analyzed with an orthogonal projection to latent structures (OPLS) regression model with SIMCA 14.0 (Umetrics AB, Sweden). A regression model was computed with the postural control variable as Y-variable (outcome variable), and the motor and sensory variables as well as age, sex, pack-years and FEV_1_% of predicted as X-variables (explanatory variables), stratified by COPD or reference. The model produces the number of components, as well as the percent of variation of the X-variables that are associated with the Y-variable (R^2^Y) and an estimation of the predictive ability of the model (Q^2^) [27]. The relationships between each of the X-variables to the Y-variables were investigated by a regression coefficient plot, expressing how strongly Y is correlated to the systemic part of the X-variables. The coefficient plot also illustrates if the correlation is positive or negative. CI ≠ 0 were considered significant.

## Results

Basic characteristics comparing the COPD and reference groups are described in Table 1. There was no difference in age between the groups. The COPD group had a higher proportion of smokers and ex-smokers as well a higher burden of tobacco smoking (pack-years) compared to the reference group. Further, any respiratory symptoms were more common in the COPD group, and individuals with COPD had significantly lower FEV_1_% of predicted as given by the inclusion-exclusion criteria in beforehand. The COPD group had lower total scores in the SPPB, and especially in regard to the gait-component.

**Table 1:** Basic characteristics of COPD and the reference group.

Characteristic	References	COPD	*p-value*
	n = 34	n = 22	
Age, mean (SD)	74.9 (4.0)	74.0 (6.2)	0.527
Women, n (%)	19 (55.9)	9 (40.9)	0.274
*Smoking habits*			
Non-smoker, n (%)	21 (61.8)	2 (9.1)	
Ex-smoker, n (%)	12 (35.3)	13 (59.1)	
Smoker, n (%)	1 (2.9)	7 (31.8)	**<0.001**
Pack years, mean (SD)	9.5 (16.2)	25.5 (17.5)	**0.001**
Height, m, mean (SD)	1.68 (0.9)	1.68 (0.9)	0.759
Weight, kg, mean (SD)	74.5 (13.3)	76.6 (15.8)	0.595
BMI, kg/m2, mean (SD)	26.2 (4.0)	27.3 (5.1)	0.392
FEV_1_% pred (SD)	94.6 (12.8)	57.2 (13.1)	**<0.001**
Any respiratory symptoms, n (%)	11 (32.4)	19 (86.4)	**<0.001**
Cardiovascular disease, n (%)	13 (38.2)	7 (31.8)	0.625
Number of prescribed medications, n (SD)	3.2 (3.0)	3.8 (2.7)	0.467
Health: better than other, n (%)	20 (58.8)	7 (31.8)	0.084
≥ 1 falls past month, n (%)	6 (17.6)	4 (18.2)	0.959
≥ 1 falls past six months, n (%)	8 (23.5)	7 (31.8)	0.494
Low physical activity, n (%)	4 (11.8)	5 (22.7)	0.275
SPPB total, median (IQR)	11.0 (11–12)	10.5 (9–11)	**0.008**
Balance, median (IQR)	4.0 (3–4)	4.0 (3–4)	0.684
Gait, median (IQR)	4.0 (4–4)	3.0 (3–4)	**<0.001**
Chair stand, median (IQR)	4.0 (3–4)	3.5 (3–4)	0.299

**Notes**: p-values<0.05 in bold. “Ex-smoker” was defined as stopped since at least 12 months. “Any respiratory symptom” was defined as at least one of persistent cough, productive cough, recurrent wheeze, or dyspnea. Cardiovascular disease: at least one of coronary heart disease, congestive heart failure or stroke. Health: better than other, self-related health score of better than others in same age. Comparing mean values with student’s t-test. Comparing proportions with Chi-2 test. Comparing median values with Mann-Whitney u-test.

**Abbreviations**: BMI, Body mass index; FEV_1_% pred, Forced expiratory volume in one second percent of predicted; IQR, Interquartile range; SD, Standard deviation; SPPB, Short physical performance battery.

### Motor and sensory systems

In the test for the vestibular system, positive nystagmus using Frenzel glasses was more common in the COPD group compared to in the reference group (31.8% vs. 2.9%, p = 0.003). The other tests of the sensory systems yielded no significant differences between the groups (Table 2). When testing muscular strength, the COPD group was weaker in the test of ankle plantar flexion than the reference group (1.24 vs 0.92, p = 0.001), but no other significant differences were identified (Table 3).

**Table 2:** Comparisons of sensory measures and reaction time between COPD and the reference group.

Variables	References	COPD	*95% CI mean difference*	*Effect size* *	*p-value*
	n = 34	n = 22			
Visual acuity, mean (SD)	0.78 (0.17)	0.80 (0.13)	-0.11–0.07	- 0.131	0.634
*Pressure sensitivity*, *median (IQR)*					
Right ankle	2 (2–4)	2 (2–2)	-	-0.010	0.926
Left ankle	2 (2–4)	2 (2–2.5)	-	-0.010	0.926
Positive nystagmus, n (%)	1 (2.9)	7 (31.8)	-	-	**0.003**
*Joint position sense AE*, *mean (SD)*					
Knee	6.05 (5.4)	5.2 (3.9)	-1.80–3.54	0.179	0.517
Ankle	4.11 (2.2)	5.2 (3.7)	-2.67–0.54	-0.365	0.190
Reaction time (ms), mean (SD)	391.6 (60.2)	382.1 (64.1)	-24.6–43.6	0.254	0.579

**Notes**: p-values<0.05 in bold

*Cohen’s d

**Abbreviations**: AE, Absolute error; IQR, Interquartile range; SD, Standard deviation.

**Table 3:** Comparisons of muscle strength between COPD and the reference group, mean of adjusted muscle strength during maximum isometric contraction.

Variables	References	COPD	*95% CI mean difference*	*Effect size* *	*p-value*
	n = 34	n = 22			
	*Mean (SE)*				
*Hand grip (Force in N* ** *)*					
HGS Left	16.6 (0.57)	15.5 (0.71)	- 0.69–2.98	0.029	0.214
HGS Right	17.5 (0.74)	16.7 (0.91)	- 1.64–3.09	0.007	0.541
*Lower limb*, left side (torque in Nm***)					
Hip extension	0.68 (0.44)	0.62 (0.55)	- 0.08–0.20	0.015	0.785
Hip abduction	0,73 (0.05)	0.67 (0.06)	-0.11–0.22	0.008	0.509
Knee extension	1.25 (0.05)	1.10 (0.07)	-0.02–0.32	0.057	0.082
Knee flexion	0.98 (0.04)	0.92 (0.05)	-0.05–0.19	0.026	0.247
Ankle dorsal flexion	0.31 (0.02)	0.28 (0.02)	-0.03–0.09	0.018	0.336
Ankle plantar flexion	1.24 (0.06)	0.92 (0.07)	-0.13–0.50	0.189	**0.001**

**Notes**: p-values<0.05 in bold, all analyzes are adjusted for age and sex.

*Partial eta squared.

**Adjusted means (Force / body weight ^0.67^)

***Adjusted means (Torque/body weight^1^)

**Abbreviations**: HGS, Hand grip strength; SE, Standard Error.

### Postural control

Individuals with COPD had larger sway compared to the reference group in the mediolateral direction in the trial with unstable surface and eyes open (ML UEO) (p = 0.014), as well as smaller amplitude in the anteroposterior limits of stability trial (AP LoS) (p = 0.019) (Table 4), both indicating worse postural control. The other postural control variables yielded no significant differences between the groups.

**Table 4:** Comparisons of postural control between COPD and reference group: Mean of adjusted maximum amplitudes during static trials and limits of stability.

	**Maximum ML amplitude**				
**Trial**	**References**	**COPD**			
	*Mean (SE) in mm* *		*95% CI of difference*	*Effect size* **	*p-value*
SEO	8.53(1.0)	10.18 (1.3)	-4.91–1.61	0.020	0.313
SEC	9.00 (0.9)	10.06 (1.1)	-3.80–1.68	0.012	0.441
UEO	17.13 (1.0)	21.30 (1.3)	-7.45 –-0.888	0.115	**0.014**
UEC	29.97 (5.0)	35.58 (6.7)	-22.61–11.40	0.009	0.511
LoS	128.5 (5.4)	125.6 (6.6)	-14.23–20.06	0.002	0.734
	**Maximum AP amplitude**				
Trial	**References**	**COPD**			
	*Mean (SE) in mm* *		*95% CI of difference*	*Effect size* **	*p-value*
SEO	16.96 (2.0)	22.64 (2.5)	- 12.26–0.899	0.055	0.089
SEC	23.80 (2.1)	26.34 (2.5)	-9.03–4.14	0.011	0.459
UEO	31.14 (1.4)	35.66 (1.8)	-9.14–0.102	0.072	0.055
UEC	54.56 (3.0)	59.19 (4.0)	-14.64–5.38	0.019	0.356
LoS	88.36 (2.5)	78.82 (3.0)	1.64–17.46	0.103	**0.019**

**Notes**: p-values<0.05 in bold, all analyzes are adjusted for age and sex.

*All amplitudes are adjusted for the participant’s height in m.

**Partial eta squared

**Abbreviations** SE, Standard error; ML, mediolateral; AP, anteroposterior; SE, standard error; SEO, stable firm surface with eyes open; SEC, stable firm surface with eyes closed; UEO, unstable soft surface with eyes open; UEC, unstable soft surface with eyes closed; LoS, limits of stability.

### Regression models

**Fig 2:**
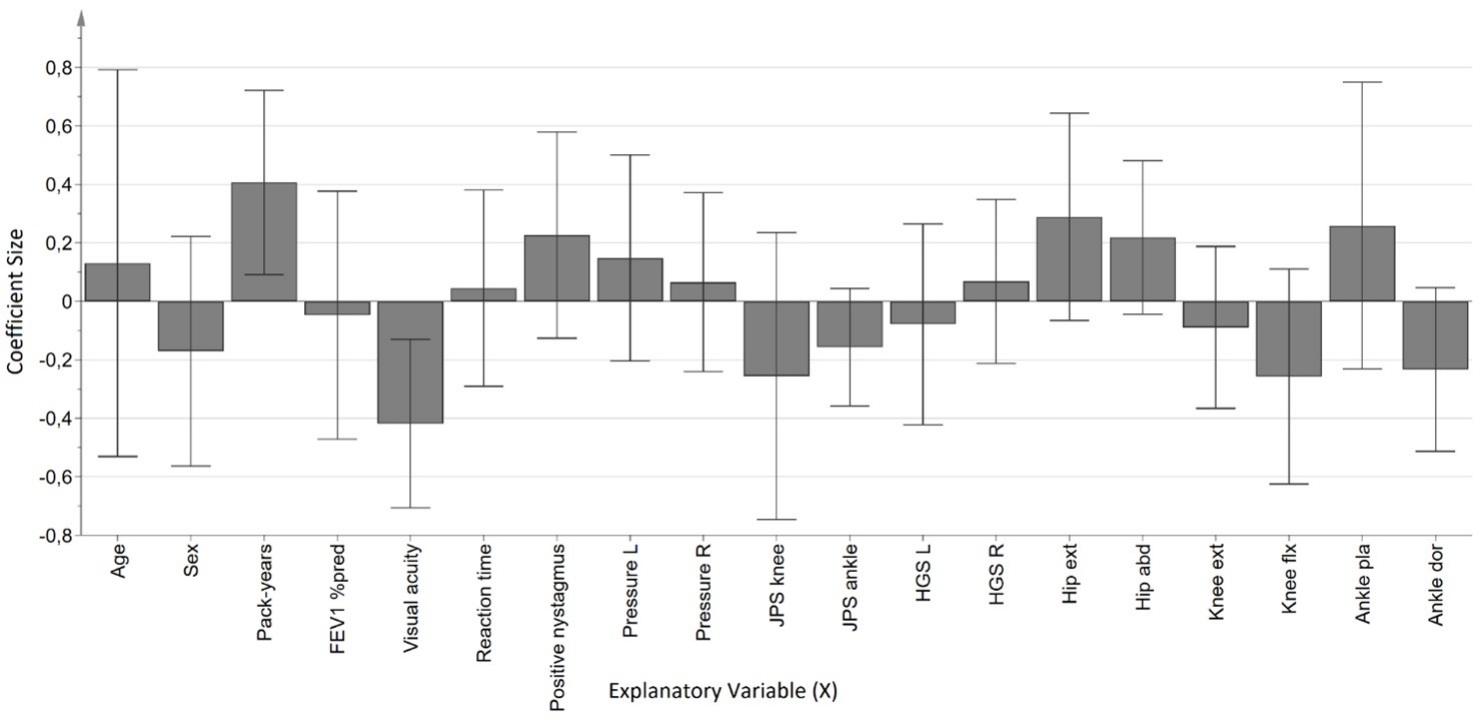
Coefficient plot of the OPLS-regression model with ML UEO (maximum mediolateral amplitude in unstable eyes open trial) as outcome, in the COPD group. **Notes**: The direction of the coefficients represent positive or negative associations with increased sway amplitude. Variables have been centered and scaled for unit variance. Error bars (95% confidence interval) not including zero indicate significant coefficients. **Abbreviations**: FEV_1_% pred, Forced expiratory volume in 1 second, percent of predicted; L, Left; R, Right; JPS, Joint position sense, HGS, Hand grip strength, Hip ext, Hip extension; Hip abd, Hip abduction; Knee ext, Knee extension, Knee flx, Knee flexion; Ankle pla, Ankle plantar flexion; Ankle dor, Ankle dorsiflexion.

Two different regression models were created with ML UEO and AP LoS as Y-variables (outcomes), respectively, stratified by COPD and reference group. The ML UEO regression model among individuals with COPD was significant and consisted of one predictive component where the X-variables explained 82.2% (R^2^Y) of the variance of ML UEO and had a predictive ability of 39.7% (Q^2^). When inspecting the coefficient plot (Fig 2), there were two significant variables; pack-years and visual acuity. A larger number of pack-years (tobacco exposure) and a worse visual acuity was associated to a larger mediolateral sway in the static trial with the unstable soft surface and eyes open. The ML UEO regression model for the reference group was not significant, and thus not presented.

**Fig 3:**
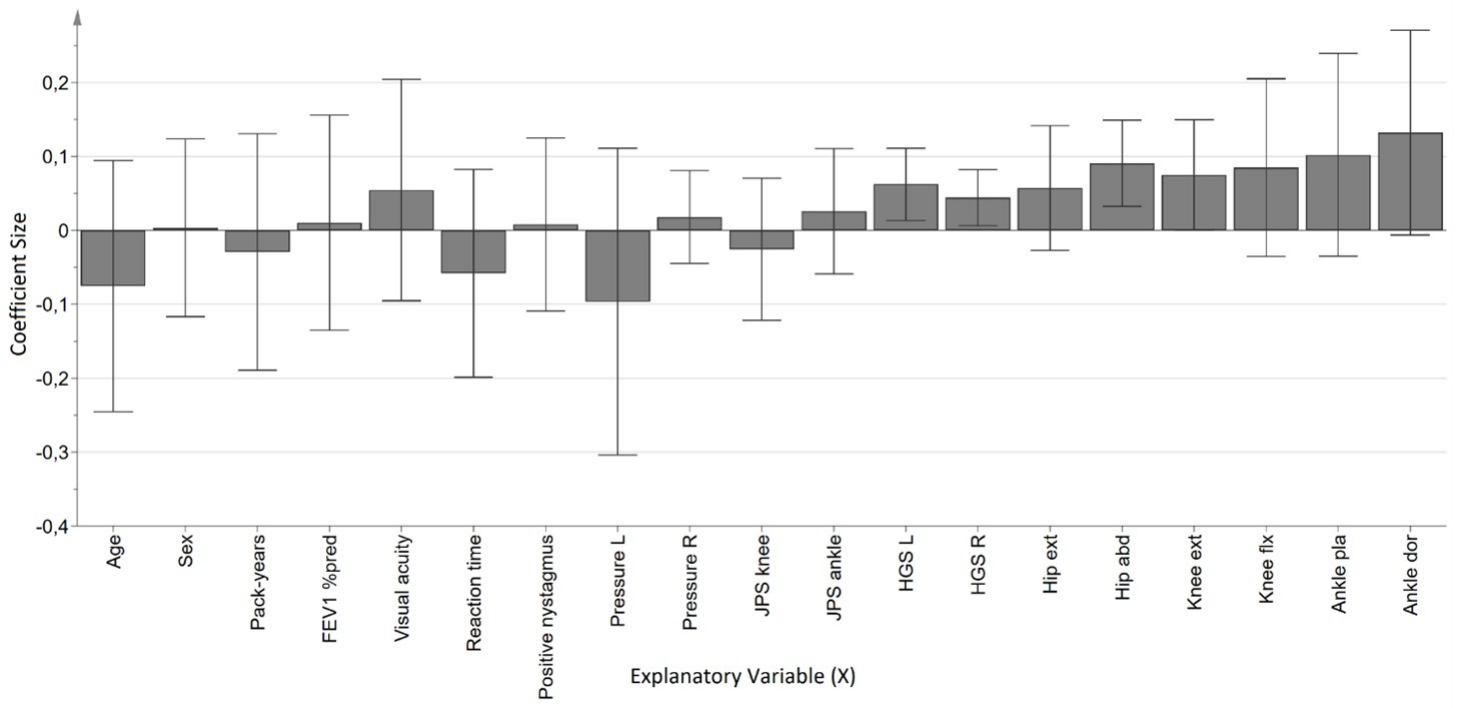
Coefficient plot of the OPLS-regression model with AP LoS (maximum anteroposterior amplitude in limits of stability test) as outcome, in the COPD group. **Notes**: The direction of the coefficients represent positive or negative associations with increased sway amplitude. Variables have been centered and scaled for unit variance. Error bars (95% confidence interval) not including zero indicate significant coefficients. **Abbreviations**: FEV_1_% pred, Forced expiratory volume in 1 second, percent of predicted; L, Left; R, Right; JPS, Joint position sense, HGS, Hand grip strength, Hip ext, Hip extension; Hip abd, Hip abduction; Knee ext, Knee extension, Knee flx, Knee flexion; Ankle pla, Ankle plantar flexion; Ankle dor, Ankle dorsiflexion.

**Fig 4:**
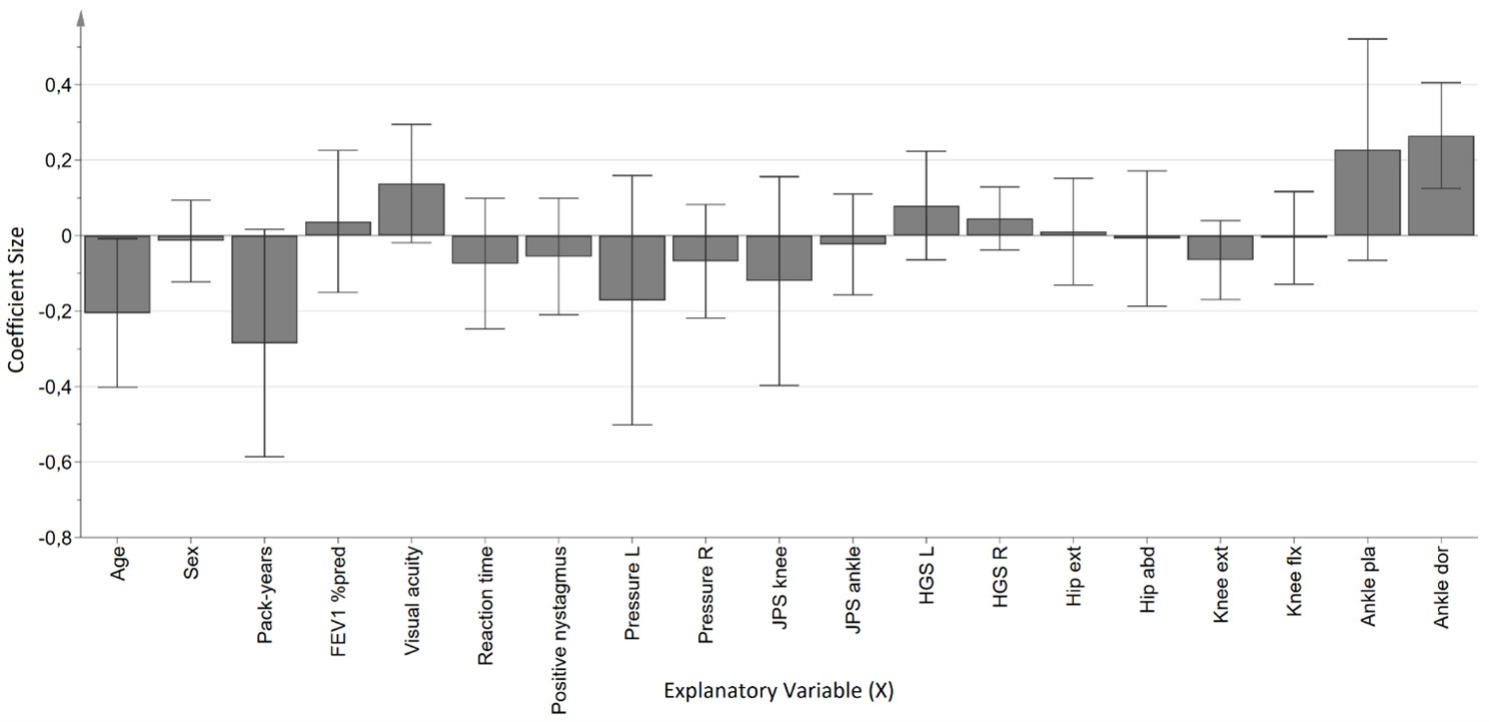
Coefficient plot of the OPLS-regression model with AP LoS (maximum anteroposterior amplitude in the limits of stability test) as outcome, in the reference group. **Notes**: The direction of the coefficients represent positive or negative associations with increased sway amplitude. Variables have been centered and scaled for unit variance. Error bars (95% confidence interval) not including zero indicate significant coefficients. **Abbreviations**: FEV_1_% pred, Forced expiratory volume in 1 second, percent of predicted; L, Left; R, Right; JPS, Joint position sense, HGS, Hand grip strength, Hip ext, Hip extension; Hip abd, Hip abduction; Knee ext, Knee extension, Knee flx, Knee flexion; Ankle pla, Ankle plantar flexion; Ankle dor, Ankle dorsiflexion.

The AP LoS regression model for the COPD group was significant, but weak and not valid, and consisted of one predictive component where the X-variables explained 35.9% (R^2^Y) of the variance of AP LoS and a predictive ability of only 8.2% (Q^2^). Four muscle strength variables were significant in the coefficient plot (Fig 3), left and right hand grip strength, knee extension and hip abduction, indicating that a higher general strength is associated with a larger AP LoS, i.e better postural control. The model for the reference group was also significant and had one predictive component where the X-variables explained 73,8% (R^2^Y) of the variance of AP LoS and had a predictive ability of 52,2% (Q^2^). The coefficients were significant for age and ankle dorsal flexion (Fig 4), indicating that lower age and higher dorsiflexion strength were associated to a higher AP LoS amplitude, i.e. better postural control in the reference group.

## Discussion

In this motion laboratory study, individuals with COPD had reduced postural control compared to an age-matched reference group without airway obstruction, significantly so in the mediolateral direction during quiet stance on an unstable soft surface with eyes open, as well as in the anteroposterior direction in limits of stability test. There was a general trend towards muscle weakness among those with COPD compared to the referents in all tested muscle groups, but only significantly so in ankle plantar flexion. In the OPLS regression model with mediolateral unstable surface eyes open (UEO) amplitude as outcome, an increased postural sway was associated with the burden of tobacco exposure and reduced visual acuity within the COPD group. In a similar model, hand grip-, hip abduction- and knee extension strength were significantly associated with the anteroposterior limits of stability (LoS). In the reference group, the corresponding model yielded significant associations between anteroposterior LoS and age and ankle dorsal flexion strength only.

### Postural control

Even though only two of ten postural control variables showed statistical differences (ML UEO & AP LoS), there was a tendency that the COPD group had a larger sway in all of the four quiet stance trials and lower amplitudes in limits of stability, indicating worse postural control. Individuals with COPD had a larger mediolateral amplitude in the UEO trial, in accordance with previous studies of individuals with severe to very severe COPD [9, 12] where mediolateral postural control was impaired compared with reference groups. Our results also showed that the COPD group had a smaller LoS amplitude (worse postural control) in the anteroposterior direction. To our knowledge, no study has previously investigated CoP limits of stability among individuals with COPD. However, the similar functional reach test [28] have previously been used to detect limitations in individuals with COPD [29], and the smaller AP LoS in our study is in line with previous research.

### Underlying systems of postural control

The regression model for ML UEO among individuals with COPD provided two significant associations, visual acuity and pack-years. The association between visual acuity and amplitude was expected since it was a trial on an unstable soft surface with eyes open, i.e. the participants could use their visual system to maintain postural control. The findings that the burden of tobacco exposure was significant, rather than severity of obstruction (FEV_1_% of predicted) was however not expected. This may be explained by the fact that smoking is a major risk factor for multiple diseases and health outcomes besides COPD [30], and the amount of pack-years [31, 32] has previously been related to neuropathy among individuals with COPD. It is possible that the finding in our study is related to a systemic effect caused directly by tobacco exposure. In the regression model we used for ML UEO as outcome, none of the muscle strength variables were significantly associated with a larger amplitude, i.e. reduced postural control among individuals with COPD. This is in accordance with previous results where even though lower muscle strength was found among patients with COPD compared to references, there was no association between muscle strength and postural control among those with COPD [8]. However, in the regression model for AP LoS in the COPD group, several muscle strength measures were significantly associated to a larger amplitude, i.e., better postural control. The corresponding model for the reference group yielded significant associations only with age and ankle dorsal flexion. The results from the OPLS-models show that quiet stance and limits of stability have different requirements of the motor and sensory systems.

Among individuals with COPD, the proportion with positive nystagmus using Frenzel glasses was higher compared to in the reference group, indicating a disturbance in the vestibular system, more specifically the vestibular-occular reflex [33]. Chronic hypoxia among patients with COPD has been speculated to affect the vestibular systems, and a previous study found general limitations in the audio-vestibular system among patients with COPD compared to references [34]. Although previous research has shown reduced postural control in more challenging tasks, which involved higher demands on the vestibular system [8], they found no clear evidence of vestibular impairments among individuals with COPD. Accordingly, the occurrence of positive nystagmus test did not yield significance in any of the OPLS-regression models in our study and there was no difference between the groups in the UEC trial which requires greater reliance on the vestibular system, and thus probably had no major impact on the observed worse postural control in the COPD group. Still, we cannot exclude that vestibular impairment is one of the mechanisms contributing to increased risk of falling among individuals with COPD and further research of this is needed. In this study, there was no difference in reaction time between the COPD and non-COPD groups, and reaction time was not significant in the OPLS-models. This is in line with a previous study that found no differences in measured muscle onset (reaction time) during stepping reactions between patients with COPD compared to references [10]. Taken together, this indicates that the speed of signal transmission is not a major factor for postural control disturbances in COPD.

There were no significant differences in ankle or knee proprioception, assessed with joint position sense test, between the groups, and neither of the variables were significant in the regression models. No previous study has investigated this before, however, previous research has found that individuals with COPD increased their dependence of ankle proprioception and decreased reliance on trunk proprioception, compared to references in postural control test using local muscle vibration [11]. Lumbar proprioception was not investigated in this study and would be relevant to include in future research.

Individuals with COPD had significantly lower muscle strength only in ankle plantar flexion compared to the reference group. However, all tested muscle groups had numerical lower values among those with COPD. Although previous studies have reported muscle weakness in COPD compared to references, it is unclear how much this contributes to postural control impairment [8, 10, 35]. A recently published review regarding the role of muscle weakness for postural control impairments in COPD concluded that current research do support an association between muscle strength and postural control, but still, there are also other underlying mechanisms [36], and further, that causality is yet to be proven. As discussed above, we did not find association between muscle strength and postural sway in quiet stance (UEO), while this was present in limits of stability (LoS AP) test. These results highlight the complexity and variety of the demands on the postural control in different tasks, in line with a recently published review and meta-analysis [37]. Together, it emphasizes the value for both clinicians and researchers of using several tests for evaluation as well as a diverse intervention for postural control improvements.

### Strengths and limitations

It is a strength that the methods of measuring postural control as well as underlying motor and sensory systems, are specific assessments of respective system. Previous research of underlying deficits in sensory systems among individuals with COPD has primary focused on postural control assessments with components that increases demand on the somatosensory-, vestibular- or visual system [8, 10]. Even though other more basic performance-based methods might be more relevant for patients with COPD in a clinical environment [36], our study has a more mechanistic perspective, striving to understand the unknown underlying factors that might contribute to the impaired postural control. This exploratory aim was investigated in this study by both the large test battery with specific motor and sensory assessments including quiet stance and LoS, as well as advanced regression modeling, which has not been done before among individuals with COPD. Besides a larger proportion of women in the reference group compared to the COPD group, the basic characteristics of the COPD and reference group were comparable regarding age, height, weight, cardiovascular diseases and number of medications.

Even though the study sample was large enough to detect differences in some of the variables regarding postural control and underlying motor and sensory systems, it might have been too small for others. The COPD group generally had lower strength and reduced postural control when inspecting the means without consideration of the p-value, and a larger sample (with higher statistical power) might have confirmed these differences between individuals with and without COPD. The generally low predictive ability (Q^2^) may also in part be derived from a low sample size, since the Q^2^ is an estimation of the predictive ability by internal cross-validation of the data. Further, even though multiple underlying systems was included in the regression models, postural control is a complex motor skill and there is a risk of residual confounding. Since the mediolateral postural control is argued to mainly depend on trunk muscles in contrast to anteroposterior control which is dependent of lower limb muscles, this could be an indication of competition of trunk muscle activity between respiration and postural control [9]. However, we did not evaluate trunk muscle strength in this study, thus the possible relationship between ML amplitude and trunk muscle strength in this sample is unknown. Lastly, due to the cross-sectional study design it is not possible to draw any conclusion on the causality of the associations in this study.

Besides a more thorough measurement of the vestibular system, future research could focus on longitudinal assessments of both postural control and underlying systems to investigate cause and effect of postural control impairments among individuals with COPD. Falls have severe negative outcomes [13] and are common especially among elderly with COPD [6]. Increased knowledge in this field is of importance and possible future clinical implications includes early identifications of individuals with increased risk of falls or specific fall prevention interventions.

## Conclusions

Individuals with COPD had a decreased postural control demonstrated both as a larger mediolateral sway amplitude in quiet stance as well as a decreased anteroposterior amplitude in limits of stability. In the COPD group, the mediolateral sway amplitude was related to impaired eyesight and the total burden of tobacco smoking, a finding not observed in the reference group. Further, the reduced limits of stability in anteroposterior direction was associated with weaker muscle strength in the COPD group, compared to higher age and weaker ankle dorsal flexion among the referents. Thus, this study implies that the amount of tobacco exposure and the level of muscle strength deficiency relate to different aspects of postural control limitations among individuals suffering from COPD. Longitudinal assessments of postural control is needed to determine causal relationships, as well as clinical implications.
